# Biofortified Yellow-Fleshed Potatoes Provide More Absorbable Zinc than a Commonly Consumed Variety: A Randomized Trial Using Stable Isotopes in Women in the Peruvian Highlands

**DOI:** 10.1016/j.tjnut.2023.08.028

**Published:** 2023-08-28

**Authors:** Reyna Liria-Domínguez, Mary Penny, Paul Antony Kroon, Gabriela Burgos, Jack Dainty, Christophe Zeder, Michael B. Zimmermann, Janet King, Richard Mithen, Erick Boy, Olla Al-Jaiballi, Susan Fairweather-Tait

**Affiliations:** 1 Instituto de Investigación Nutricional, Lima, Peru; 2 Food, Microbiome and Health Program, Quadram Institute Bioscience, Norwich Research Park, Norwich, United Kingdom; 3 Genetics, Genomics, and Crop Improvement Division, International Potato Center, Lima, Peru; 4 Norwich Medical School, University of East Anglia, Norwich, United Kingdom; 5 ETH Zürich, Laboratory of Human Nutrition, Department of Health Sciences and Technology, Institute of Food, Nutrition, and Health, Zurich, Switzerland; 6 Department of Nutritional Sciences and Toxicology, University of California-Berkeley, Berkeley, CA, United States; 7 Liggins Institute, Waipapa Taumata Rau – The University of Auckland, Auckland, New Zealand; 8 HarvestPlus Programme, Innovation, Policy and Scaling Unit, International Food Policy Research Institute, Washington, DC, USA

**Keywords:** zinc absorption, biofortified crops, potatoes, stable isotopes

## Abstract

**Background:**

Zinc-biofortified potatoes have considerable potential to reduce zinc deficiency because of their low levels of phytate, an inhibitor of zinc absorption, and their high consumption, especially in the Andean region of Peru.

**Objectives:**

The purpose of this study was to measure fractional and total zinc absorption from a test meal of biofortified compared with regular potatoes.

**Methods:**

We undertook a single-blinded randomized crossover study (using ^67^Zn and ^70^Zn stable isotopes) in which 37 women consumed 500-g biofortified or regular potatoes twice a day. Urine samples were collected to determine fractional and total zinc absorption.

**Results:**

The zinc content of the biofortified potato and regular potato was 0.48 (standard deviation [SD]: 0.02) and 0.32 (SD: 0.03) mg/100 g fresh weight, respectively. Mean fractional zinc absorption (FZA) from the biofortified potatoes was lower than from the regular potatoes, 20.8% (SD: 5.4%) and 25.5% (SD: 7.0%), respectively (*P* < 0.01). However, total zinc absorbed was significantly higher (0.49; SD: 0.13 and 0.40; SD: 0.11 mg/500 g, *P* < 0.01, respectively).

**Conclusions:**

The results of this study demonstrate that biofortified potatoes provide more absorbable zinc than regular potatoes. Zinc-biofortified potatoes could contribute toward reducing zinc deficiency in populations where potatoes are a staple food.

This trial was registered at clinicaltrials.gov as NCT05154500.

## Introduction

Zinc deficiency is common in populations that consume diets with a low content of animal products and a high consumption of vegetables and cereal crops. Animal foods make an important contribution to zinc requirements because they contain highly bioavailable zinc, whereas the zinc in some cereals and vegetables may have lower absorption because of the presence of phytate. Child undernutrition is persistently high in the central Andes of Peru, and numerous smallholder families fail to meet their basic needs of energy, iron and zinc [1].

Although various strategies, including agricultural diversification, may have the potential to alleviate micronutrient deficiencies, cultural and economic factors may limit access to animal source foods and low-phytate staple foods, and so the introduction of zinc-biofortified crops could be an effective and sustainable alternative approach when they are part of the usual diet [2]. The zinc content of plants depends on environmental conditions and plant genetics [3]. Biofortification of foods can be achieved through 3 main approaches, namely conventional, agronomic, and transgenic, that involve the use of crop breeding, fertilization strategies, and biotechnology, respectively. Crop plants containing higher levels of micronutrients can be obtained via genetic modification/precision breeding or conventional plant breeding. Alternatively, for minerals, such as zinc and iron, an additional target is to reduce the phytate content to increase the absorption of these minerals [4]. In all cases, the overall goal is to increase the amount and the bioavailability of nutrients, with the objective of reducing nutritional deficiencies [5].

According to the FAO, after rice and wheat, potato was the most consumed food worldwide in 2019, with an average consumption of 66 kg/per capita/y [6], making it a good candidate crop for biofortification. It is a very well-accepted food that is consumed by all age groups and is an accessible food for the most vulnerable groups [7]. Potato is a mainstay of agriculture and diets in the high-altitude food systems of Peru, where farmers grow diverse varietal portfolios [8]. In a study conducted in 6 communities of Huancavelica, potato was the principal food staple. In populations living in extreme poverty in rural areas of the Peruvian Andes, the average potato consumption is around 600–800 g/d for women of reproductive age and 165–200 g/d for children aged between 6 and 36 mo. Considerable differences were found between communities with the average daily potato intake of women during the period of abundance in one community being as high as 1348 g/d.

The International Potato Center (CIP) has developed zinc-biofortified potatoes using conventional breeding. As potatoes contain low levels of phytate [8], zinc absorption from potatoes might be relatively high, but human studies are needed to test this hypothesis. Zinc-biofortified potatoes have the potential to contribute to a reduction in zinc deficiency, especially in populations where potatoes are an important part of the usual diet, such as in the Andean Highlands [8]. Therefore, the aim of this study was to determine fractional and total zinc absorption in test meals of zinc-biofortified potatoes in comparison with regular potatoes by conducting a single-blinded randomized crossover study.

## Materials and Methods

### Study design

The study was a randomized crossover single-blinded study with biofortified and regular potatoes in women living in the city of Huancavelica, Peru. Women aged 18–45 y that met the following criteria were included: BMI 18.5–25 kg/m2; able to come to the study center to eat the prepared potatoes on 2 occasions with a washout in between 30 d, and to remain in the study center for 7–8 h and to comply with all the study procedures; and able to understand and sign the consent form. We excluded subjects according to the following criteria: severe anemia (hemoglobin [Hb] <80 g/L adjusted for altitude) [9]; C-reactive protein (CRP) >5 mg/L; gastrointestinal or renal problems; diabetes; hypertension; pregnant (positive urine test); currently breastfeeding; taking medication, antibiotic, or supplement that could affect zinc metabolism; unwilling to discontinue zinc supplementation for ≥2 wk before participating in the study; significant blood loss in the last 6 mo due to trauma, surgery, or blood donation; and unable to eat all the potato.

### Sample size calculation

Assuming a SD of 0.2 (SD of the log-transformed) difference within pairs from a similar previous zinc study [10], a type 1 error of 5% (2-tailed) and a power of 80%, a difference in the FZA of 32% can be detected with a sample size of 24 participants. To allow for potential dropouts, we enrolled 40 subjects.

### Potato samples and meal preparation

The regular non-biofortified commercial potato variety “Amarilla” (yellow) and the zinc-biofortified yellow potato CIP311623.75 were grown in Cacara, Paucartambo, Pasco, Perú. The potatoes were harvested in May 2021 and were transported to Lima where the test meals were prepared in June–July.

For the preparation of the potato meals, the potatoes were washed and scrubbed using a brush to remove any earth that might contaminate the potato. The potatoes were boiled for ∼60 min, after that those potatoes were peeled (including removal of the eyes), mashed, and homogenized. Portions of 250 g were aliquoted into labeled plastic bags. This procedure was undertaken before the beginning of the study and all the prepared cooked potato meals were stored at −18°C in individually labeled packets. Finally, the frozen, prepared potatoes were transported from Lima to Huancavelica.

### Test meal analyses

Analyses were carried out on freeze-dried potatoes. Subsamples were ground in a stainless-steel mill only used for potato samples to avoid contamination. Zinc concentration was measured in samples digested by refluxing with HNO_3_ on a hot plate for 2 h and analysis was done by Inductively Coupled Plasma Mass Spectrometry (Thermo Fisher Scientific iCAP-Q; Thermo Fisher Scientific) at the CIP [11]. Phytic acid (PA) concentrations were measured at Swiss Federal Institute of Technology (ETH) using a modification of the Makower method [12], as described previously by Paganini et al. (2017) [13]. Individual polyphenol concentration was determined in aqueous methanol extracts using an ultraperformance liquid chromatography with mass spectrometry method [14]. Ascorbic acid was analyzed in the fresh samples by UV/Visible spectroscopy (UV 160A; Shimadzu Corp) at CIP [15].

### Screening and study procedures

In response to advertisements, people expressing an interest in the study were provided with further information (Participant Information Sheet) and their potential involvement was discussed with a member of the study team. Participants who remained interested in taking part in the study were asked to complete and sign the consent form.

Once informed consent had been obtained, the body height and weight of the participants were measured using standardized equipment and the BMI was calculated to determine if participants were eligible for the study. Venous blood samples (∼3 mL in a Vacutainer SST II Advances Tubes—with an inert gel barrier, and 3 mL in a K-EDTA Vacuum tube) were taken and used to measure high-sensitivity CRP (Turbidimetry, Vitros 4600—Ortho Clinical Diagnostics) and hemoglobin (5-part differential flow, Nihon Kohden), respectively. The tubes with the inert gel barrier were centrifuged and 0.5-mL aliquots of serum were transferred into cryovials. All samples were stored at <18°C until analysis. A 30-mL urine sample was collected from all candidates as part of screening to rule out pregnancy. According to the results of screening, and after checking that they complied with all the selection criteria, they were invited to participate in the study.

Women were randomly allocated to start the study with one of the varieties of potato (biofortified, meal A, or regular potato, meal B). Each type of potato had a group code so that the participants were blinded to the variety they received. A list of random numbers was prepared in Excel to establish the order of assignment to each participant. This list was drawn up before the start of the trial.

All the participants were given both types of potato in a random order, thus each participant acted as their own control. The first variety of potatoes was given on day 1 and the second on day 29. All meals were consumed alone, without other food, with drinking water. Participants were allowed to add salt to their meals to their liking. The subjects consumed 1000-g potato in 2 meals of 500 g administered on 2 separate occasions within the day, as giving the whole quantity in 1 meal would be unacceptable to most participants. The first meal was given in the morning after an overnight fast (≥8 h without food and 6 h without any drink) and in 2 portions (250 g each). When they had eaten the first half of the meal, depending on the potato variety that they had been allocated, they received 0.25-mg ^67^Zn diluted in 50-mL water. Two hours after the first portion of potato was eaten, they received an intravenous infusion of 0.2-mg ^70^Zn diluted in 50 mL of saline. Two hours after this, they received the second portion of the potato, following the same procedures. For 3 h after the second portion of potato was consumed, the subjects were not allowed to consume any food or liquid, except for the water they were given. All participants received ≥1 glass of water of 200 mL with the first portion of potato and another glass with the second portion and, those who requested a further drink were given additional water. We ensured that the subjects ate all the food offered each day.

### Stable isotopes tracers

The zinc isotopes, ^67^Zn (isotopic enrichment 90.6%) and ^70^Zn (isotopic enrichment 98.8%), were purchased as zinc oxide from Chemgas. The ^67^Zn-labeled zinc sulfate for oral administration was prepared by dissolution of the ^67^Zn oxide in an equimolar amount of diluted sulfuric acid. The resulting solution was diluted to a concentration of 0.25 mg Zn/g with water. The solution was portioned into 2 mL individual doses into polytetrafluoroethylene-PFA vials. Each vial contained 0.5-mg zinc tracer, sufficient to label 2 meals of each variety of potato for each participant. The ^70^Zn-labeled zinc chloride for intravenous administration was prepared by dissolution of the ^70^Zn oxide in a stoichiometric amount of diluted hydrochloric acid. The resulting solution was diluted to a concentration of 22 μg Zn/g with sterile physiologic saline, and the pH was adjusted to 6.0 with a 1 M sodium bicarbonate solution. The solution was sterilized by filtration and portioned into 10 mL glass vials by the pharmacy of the University Hospital of Zurich (KAZ) in accordance with Good Manufacturing Practice (GMP). A subset of the prepared vials was used to check the solution for sterility and pyrogens. During the study, 9 mL of the solution was administered, corresponding to 0.2-mg zinc. The isotopic composition of the labels was measured by Multicollector Inductively Coupled Plasma Mass Spectrometry (Neptune, Thermo Fisher Scientific) and zinc concentration was measured by inverse isotopic dilution mass spectrometry.

### Blood collection and analysis

**FIGURE 1:**
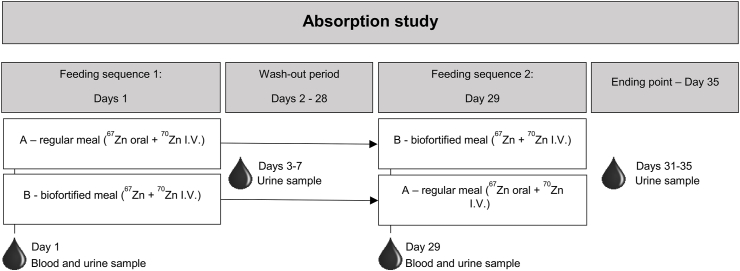
Study design to evaluate the bioavailability of zinc from potatoes (A and B refer to the potato variety: biofortified compared with nonfortified).

Fasting blood samples were collected at screening, day 1 and day 29 (Figure 1). Plasma samples were shipped from Lima to ETH Zurich on dry ice and stored at −20°C until analysis. CRP was measured using an automated immunoassay analyzer (Immulite 1000, Siemens Healthineers). Accuracy was checked by measuring control samples (3 levels) from the same manufacturer. Plasma zinc concentration was measured by inductively coupled mass spectrometry (iCap RQ, Thermo Fisher Scientific). After thawing, 200-μL plasma was pipetted in duplicate into 15 mL polypropylene tubes, and 4.8-mL diluent solution comprised of 0.5% nitric acid, 1% 2-propanol, and 0.05% Triton X-100 in ultrapure water was added [16]. The zinc calibrators were prepared in the same diluent solution. Control samples (Seronorm Trace Element Serum L-1 and L- 2, Sero AS) were measured with every run of samples to assess accuracy. The cut-off to determine zinc deficiency was 660 μg/L in plasma [17].

### Urine collection and analysis

To measure zinc absorption, we collected 100-mL urine samples at the start of the study and for 5 d after they had eaten each variety of potato: day 1 (basal, before eating the first potato variety), days 3–7, day 29 (baseline of the second potato variety), and days 31–35. Urine samples were collected in the morning (between 7 and 12) and stored frozen at −18°C (Figure 1). The urine samples were stored at ETH Zurich at −20°C until processing. The samples were thawed, transferred to 150-mL Erlenmeyer flasks, and evaporated to near dryness at 115°C overnight, under a laminar flow hood. After cooling down to room temperature, 5 mL of 65% nitric acid was added to all samples. A total of 2-mL predigested samples were then mineralized in duplicate in a microwave autoclave (TurboWave, MLS). The zinc present in the mineralized samples was isolated by anion exchange chromatography [10]. The isotopic composition of the isolated zinc was measured by Multicollector Inductively Coupled Plasma Mass Spectrometry (Neptune, Thermo Fisher Scientific).

### Calculation of fractional absorption

The fractional absorption of the ^67^Zn label from the test meal was calculated using the oral to i.v. tracer ratio method, according to principles described by Friel et al. [18]. The isotopic composition measured on day 29 samples for each participant was used as baseline isotopic composition for the second part of the study. Fractional absorption was calculated for each sample, and the mean of the 3 values of each treatment was used for the evaluation.

### Ethical approval

The protocol was approved by the University of East Anglia, Faculty of Medicine and Health, Research Ethics Committee in Norwich (United Kingdom) in December 2019 (2019/20-018) and the Ethics Committee of the Instituto de Investigación Nutricional in Peru in February 2020 (391-2020/CIEI-IIN).

All participants gave written informed consent. The study was registered at www.clinicaltrials.gov (identifier number NCT05154500).

### Statistical analysis

Calculations and statistical analyses were performed using R version 4.1.2 (R Foundation for Statistical Computing, Vienna, Austria). Normally distributed data tested by Shapiro–Wilk are reported as mean ± SD if normally distributed or as median (with interquartile range) if not normally distributed. FZA and total absorbed zinc (TAZ) values are reported as geometric means (lower SD and higher SD). Potential carryover and meal sequence effects were investigated with a mixed effects model (subjects as the random effect) using the “lmer” function in R. The significance (*P* value) of the interaction of “potato meal” (either biofortified or non-biofortified) and the “meal sequence” (either first or second) was used to test whether a carryover effect was present in the results. The *P* value of the fixed effect of “meal sequence” was used to test for a sequence or order effect. A 2-sided paired *t*-test was used to compare the general characteristics of the participants, and FZA and TAZ. *P* values 0f <0.05 were considered significant. The association (Pearson correlation) between FZA and plasma zinc concentration was examined using the “cor.test” function in the Base package of R (R Core Team [2022]). R: A language and environment for statistical computing. R Foundation for Statistical Computing, https://www.R-project.org/.)

## Results

### Nutritional composition of the potatoes

The analysis was performed 2 wk before the preparation of the test meals on a subset of the tubers used for the test meals. The test meals themselves were not analyzed. As shown in Table 1, the mean concentration of zinc in the biofortified potatoes was higher than in non-biofortified potatoes (0.48 ± 0.02 mg/100 g compared with 0.32 ± 0.03 mg/100 g, respectively), and the PA was lower (22.6 ± 0.69 compared with 42.1 ± 2.7 mg/100 g, respectively). The PA:zinc molar ratio was almost 3 times higher in the regular than the biofortified potatoes (13.45 ± 0.84 compared with 4.66 ± 0.14, respectively).

**TABLE 1:** Composition of the potato test meals (mean value ± SD, mg/100 g fresh weight) and quantity of potato eaten (g/d)

	Regular	Biofortified	*P* value
Zn	0.32 ± 0.03	0.48 ± 0.02	0.001
Phytic acid (PA)	42.1 ± 2.7	22.6 ± 0.69	<0.001
Total polyphenols	22.7 ± 0.31	25.0 ± 3.54	0.328
Tryptophan	6.92 ± 0.10	8.30 ± 1.59	0.207
Chlorogenic acid	12.61 ± 0.17	15.15 ± 1.72	0.064
Neochlorogenic acid	1.86 ± 0.05	1.06 ± 0.14	<0.001
Cryptochlorogenic acid	0.40 ± 0.01	0.14 ± 0.05	<0.001
Caffeic acid	0.68 ± 0.02	0.32 ± 0.05	<0.001
Ferulic acid	0.02 ± 0.01	0.01 ± 0.00	0.116
Rutin	0.21 ± 0.01	0.01 ± 0.00	<0.001
Ascorbic acid (mg)	9.59 ± 0.39	5.55 ± 0.51	<0.001
PA:Zn molar ratio	13.45 ± 0.84	4.66 ± 0.14	<0.001
Total weight of potato consumed (g)	1003.1 ± 4.9	1003.2 ± 5.1	
Total Zn consumed (mg)	3.21 ± 0.27	4.85 ± 0.21	0.001

### Study participants

**FIGURE 2:**
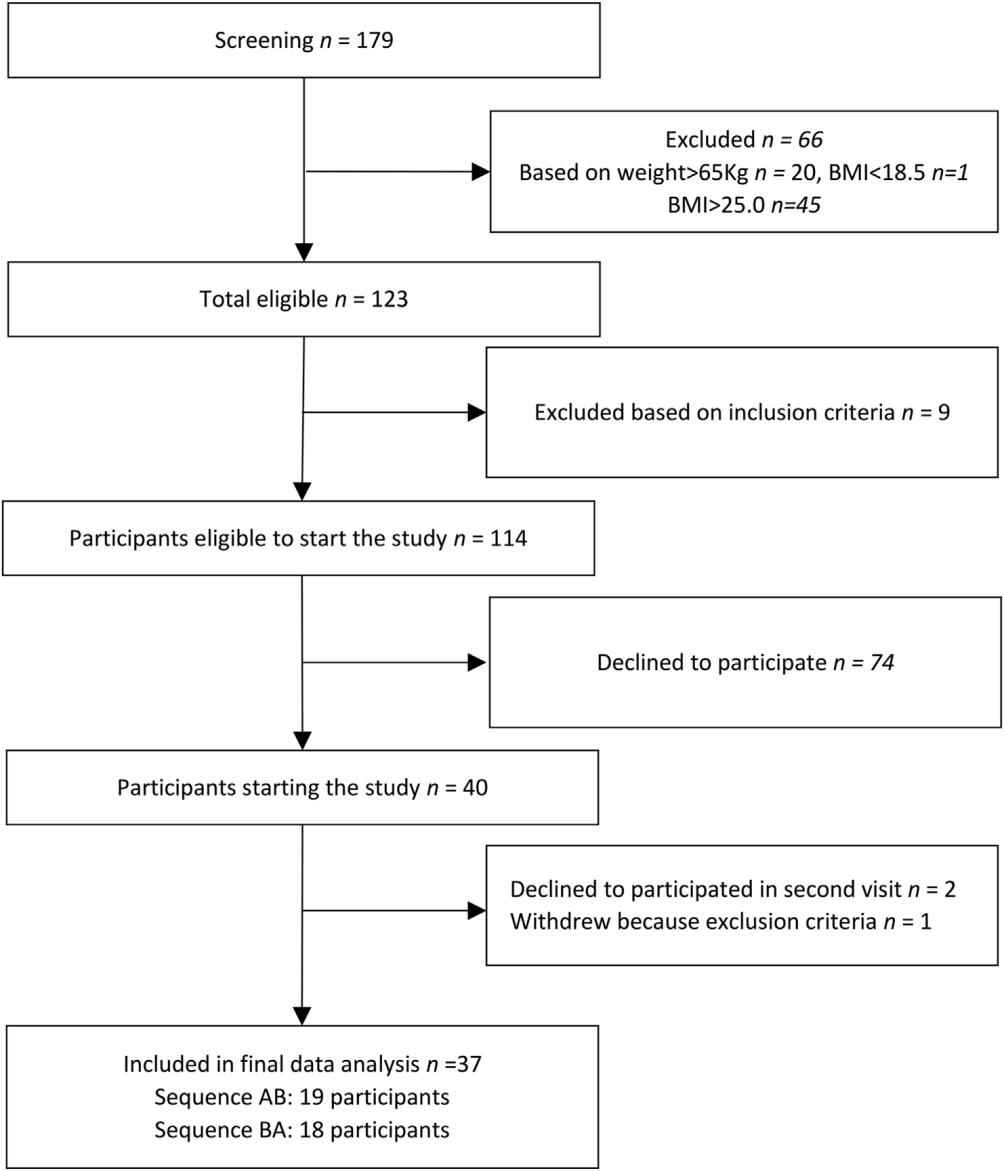
Study overview diagram.

A total of 40 participants were recruited for the study and 37 completed the study in accordance with the protocol (Figure 2). Two participants withdrew after the first phase of the study. One participant withdrew during the second phase of the study. None of the participants showed an elevated CRP concentration (>5 mg/L) at either sampling time. A total of 37 participants completed the study (18 were randomly assigned to potato sequence AB and 19 were assigned to sequence BA). The mean age of the women who completed the study was 23.0 ± 3.9 y and mean BMI was 22.3 ± 1.5 kg/m^2^, mean plasma zinc was 756 ± 113 μg/L (Table 2). According to the criteria set by the International Zinc Nutrition Consultative Group (<700 μg/L plasma zinc) [19], 29.7% of the women were zinc deficient. The quantity of potato eaten (per day) was just over 1000 g for both types of potato (Table 1).

**TABLE 2:** Characteristics of participants

Parameter	Enrolled	Day 1 (baseline)
Participants (*n*)	40	37
Age (y)^1^	23.3 ± 4.58	23.0 ± 3.9
Height (cm)^1^	153.2 ± 4.7	152.8 ± 4.8
Weight (kg)^1^	52.2 ± 4.8	52.0 ± 4.7
BMI (kg/m^2^)^1^	22.3 ± 1.6	22.3 ± 1.5
Plasma zinc (μg/L)^1^	754 ± 109	754 ± 140
Zinc deficient^2^	11 (27.5%)	11 (29.7%)
C-reactive protein (mg/L)^1^	1.58 ± 1.35	1.00 ± 1.26

1 Mean ± SD.

2 *n* (%). Cut-off for zinc deficiency <700 μg/L [20].

### Mass spectrometric measurements

The ^66^Zn/^67^Zn and ^66^Zn/^70^Zn isotopic ratios measured in the urine samples following tracer administration all showed measurable isotopic enrichments compared with the baseline samples. The isotopic enrichments ranged from 1.6‰ to 37.8‰ for ^66^Zn/^67^Zn and from 42‰ to 190‰ for ^66^Zn/^70^Zn. The limit of detections of isotopic enrichments was 0.10‰ for ^66^Zn/^67^Zn and 0.34‰ for ^66^Zn/^70^Zn, respectively.

### FZA and TAZ

**FIGURE 3:**
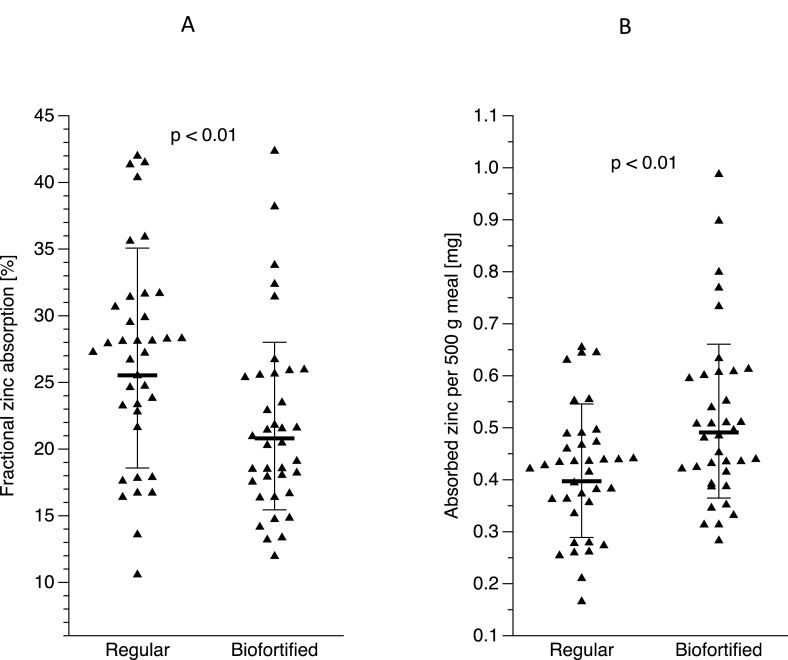
Fractional zinc absorbed (%) (A) and total absorbed zinc per 500-g meal (mg) (B) of regular and biofortified potatoes. Isotopic administration: ^67^Zn-labeled zinc sulfate for oral administration and ^70^Zn-labeled zinc chloride for intravenous administration. Values are estimated means ± SEM. *n* = 37.

The mixed effects model results for the interaction of “potato meal” and “meal sequence” was not significant (*P* = 0.779), which indicates that carryover effects were unlikely to be present. There was no effect of meal sequence either (*P* = 0.198). The geometric mean FZA was significantly lower (*P* < 0.01) for the biofortified than for the non-biofortified variety, 20.8% (–SD 15.5% and +SD 28.1%) and 25.5% (–SD 18.5% and +SD 35.1%), respectively. The total amount of zinc absorbed from a 500 g potato meal was 0.49 mg (0.36–0.66 mg) for the biofortified variety and 0.40 mg (0.29–0.55 mg) for the regular variety. On average, 22.5% more zinc was absorbed from the biofortified variety than from the regular variety (*P* < 0.01), for the same amount of potato (Figure 3). The FZA of biofortified potatoes compared with plasma Zn had a correlation coefficient of −0.26 but was not significantly correlated (*P* = 0.128). The FZA of regular potatoes compared with plasma Zn had a correlation coefficient of −0.25 but was not significantly correlated (*P* = 0.134).

## Discussion

The zinc-biofortified potato contained ∼50% more zinc than the traditional variety; and, despite lower FZA from the biofortified potato, TAZ was 22.5% greater. A saturable dose–response relationship has been described for zinc, with a predicted maximum zinc absorption of 13 mg [20]. The quantity of zinc in the potato test meals is <3 mg, so they are well within the linear part of the dose–response curve, and therefore the higher FZA from the regular potato meal is likely to be the result of the lower dose.

Given the low levels of PA in potatoes, zinc in potatoes could have a higher bioavailability than that in cereals and legumes. PA chelates zinc in the gut and is the chief zinc bioavailability antagonist [21]. The biofortified and regular potatoes used in this study have a PA level of 22 and 42 mg/100 g, values that are significantly lower than those reported for biofortified beans (1483–2100 mg/100 g), for low-PA beans (around 105 mg/100 g) [22], and for zinc-biofortified rice (365–761 mg/100 g) [23].

The presence of PA in plant foods will inhibit the absorption of zinc, depending on the level of PA and the PA:zinc molar ratio. According to the Miller equation [20], molar ratios (PA:Zn) above 15:1 will inhibit zinc absorption and are associated with suboptimal zinc status in humans with a critical ratio of 18:1 [24]. The PA:zinc molar ratios for the regular potato meal and the biofortified potato meal were 13.45 and 4.66, respectively.

The FZA from the meal prepared with zinc-biofortified potatoes (20.8%) was lower than the meal prepared with regular potatoes (25.5%). These values are very much higher than FZA from a 300 g meal of porridge prepared with 50-g biofortified wholewheat flour (total zinc content 3.6 mg, FZA 6.3%) and of a hydroponically biofortified wheat porridge served as chapattis with a cauliflower–potato sauce (total zinc content 4.0 mg, FZA 5.7%) [10]. Although the potato meals contain less zinc than the biofortified wheat meal (1.85 and 2.67 mg for the 500 g of regular and biofortified meal, respectively), and are not directly comparable, it appears that zinc in potatoes is considerably more bioavailable than biofortified wheat.

Taking into consideration the mean intake of potatoes in women of childbearing age from the same urban highland area of study (181 g/d) (unpublished data from 86 women of childbearing age, Liria-Domínguez MR), the biofortified potato meal would contribute 13% of the estimated average requirement (EAR) for zinc for women of childbearing age [24]. However, there is a high potato consumption of populations living in extreme poverty in rural areas of the Peruvian Andes; the average potato consumption is around 600–800 g/d for women of reproductive age and 165–200 g/d in children aged between 6 and 36 mo. Therefore, the contribution of biofortified potato to zinc requirements would be considerable (between 42% and 56% of the EAR for women of reproductive age and between 31% and 38% of the EAR in children aged from 6 to 36 mo) [24].

The concentration of PA in the biofortified potatoes used in this study was about half that found in regular potatoes; a 500 g meal contained 113-mg PA compared with 210 mg in a 500 g meal of regular potatoes. These levels are low compared with daily intakes of 300–800 mg reported for mixed diets [25]. However, the PA:zinc molar ratio is the key moderator of zinc absorption. PA:zinc molar ratios of 12–15 or higher have been shown to reduce dietary zinc absorption by ∼50% [25,26]. The PA:zinc molar ratio was 4.66 in the biofortified potatoes, compared with 13.45 in regular potatoes. WHO classified a diet of high zinc bioavailability as having a PA:zinc ratio <5 [26]; therefore, it is likely that there was a modifying effect of PA on zinc absorption from regular potatoes but not in the biofortified potatoes.

Previous studies on biofortified food crops have evaluated the bioavailability of zinc in cereals and beans [10,22,23] but to our knowledge, this is the first study measuring zinc absorption from potatoes. The strength of the study is that we used a well-established stable isotope technique to measure zinc absorption to assess zinc bioavailability from the 2 varieties of potato. The results obtained in this study are a prerequisite for further research on biofortified potatoes, in particular, efficacy trials investigating changes in zinc status with long-term intake of zinc-biofortified potatoes. A weakness is that the findings are restricted to communities where potato is a major component of the diet, namely in rural areas of Latin American highlands. Zinc absorption in meals that combine potatoes with other foods such as beans, might produce different findings depending on the concentration of phytate as this reduces zinc bioavailability.

Our study shows that biofortified potatoes supply a higher quantity of absorbable zinc than regular potatoes. The advantage of potatoes is that they have a relatively low-phytate content, their consumption is widespread, the quantity of potatoes eaten daily is high, and they have a relatively low cost and high acceptability. Physiologic requirements for zinc can be difficult to meet, especially in low-income populations with a limited variety of foods in their diet [27]. In the Andean rural population, if current potato varieties could be replaced by biofortified potatoes, this could provide more dietary zinc and reduce zinc deficiency. Future research is needed to assess the long-term impact of consuming biofortified potatoes on zinc nutrition in populations with marginal zinc intakes.

## Author contributions

The authors’ responsibilities were as follows – GB, RL-D, SF-T, MBZ, CZ: conceived the study; RL-D, GB, CZ: conducted the study and analyzed the data; RL-D: wrote the manuscript; and all authors: contributed to the design of the study and read and approved the final manuscript.

## Conflict of interest

The authors report no conflicts of interest.

## Funding

This research was funded by the Biotechnology and Biological Sciences Research Council (BBSRC) through a Global Challenges Research Fund (GCRF) Grant (BB/S014039/1) and via the Institute Strategic Programme Grants ‘Food Innovation and Health’ (BB/R012512/1) and its constituent project BBS/E/F/000PR10343 and ‘Food Microbiome and Health’ (BB/X011054/1) and its constituent project BBS/E/F/000PR13630. We gratefully acknowledge USAID Feed the Future Crops to End Hunger award to CIP (DIS-B-AID-BFS-IO-17-00005). For the purpose of open access, the authors have applied a Creative Commons Attribution (CC BY) license to any Author Accepted Manuscript version arising.

## Data availability

Data described in the manuscript, code book, and analytic code will be made available upon request with an embargo end date of 9 mo.
